# A Novel Mouse Model of Intrahepatic Cholangiocarcinoma Induced by Azoxymethane

**DOI:** 10.3390/ijms241914581

**Published:** 2023-09-26

**Authors:** Yohei Shirakami, Junichi Kato, Masaya Ohnishi, Daisuke Taguchi, Toshihide Maeda, Takayasu Ideta, Masaya Kubota, Hiroyasu Sakai, Hiroyuki Tomita, Takuji Tanaka, Masahito Shimizu

**Affiliations:** 1 Department of Gastroenterology, Gifu University Graduate School of Medicine, Gifu 501-1194, Japan; yfa52710@nifty.com (J.K.); om19840905@gmail.com (M.O.); fiction_may_relieve@yahoo.co.jp (D.T.); toshi_z218@yahoo.co.jp (T.M.); taka.mailbox.789@gmail.com (T.I.); kubota.masaya.d5@f.gifu-u.ac.jp (M.K.); sakai.hiroyasu.a8@f.gifu-u.ac.jp (H.S.); shimizu.masahito.j1@f.gifu-u.ac.jp (M.S.); 2 Department of Tumor Pathology, Gifu University Graduate School of Medicine, Gifu 501-1194, Japan; tomita.hiroyuki.y6@f.gifu-u.ac.jp; 3 Department of Pathological Diagnosis, Gifu Municipal Hospital, Gifu 500-8513, Japan; tmntt08@gmail.com

**Keywords:** intrahepatic cholangiocarcinoma, steatohepatitis, fatty liver, azoxymethane, liver cancer, leptin

## Abstract

Cholangiocarcinoma is the second most common primary cancer of the liver and has a poor prognosis. Various animal models, including carcinogen-induced and genetically engineered rodent models, have been established to clarify the mechanisms underlying cholangiocarcinoma development. In the present study, we developed a novel mouse model of malignant lesions in the biliary ducts induced by the administration of the carcinogen azoxymethane to obese C57BLKS/J-db/db mice. A histopathological analysis revealed that the biliary tract lesions in the liver appeared to be an intrahepatic cholangiocarcinoma with higher tumor incidence, shorter experimental duration, and a markedly increased incidence in obese mice. Molecular markers analyzed using a microarray and a qPCR indicated that the cancerous lesions originated from the cholangiocytes and developed in the inflamed livers. These findings indicated that this is a novel mouse model of intrahepatic cholangiocarcinoma in the context of steatohepatitis. This model can be used to provide a better understanding of the pathogenic mechanisms of cholangiocarcinoma and to develop novel therapeutic strategies for this malignancy.

## 1. Introduction

Cholangiocarcinoma (CCA) is the second most common primary cancer of the liver after hepatocellular carcinoma (HCC) and is the most common cancer of the biliary duct [1]. Although CCA is relatively rare, comprising approximately 3% of all gastrointestinal cancers, it is considered aggressive and has a poor prognosis, with a 5-year overall survival rate of less than 8% [2,3,4]. For a long time, to treat CCA with chemotherapeutic agents, cytotoxic chemotherapy, particularly a combination of gemcitabine and cisplatin, has been used as the first-line therapy. Recently, several molecular targeted drugs for CCA, including the programmed death-ligand 1 immunotherapy durvalumab and the fibroblast growth factor receptor (FGFR)-targeting pemigatinib and futibatinib, have been developed and approved [5,6,7], which has contributed to improved prognosis; however, there is still a critical need to establish additional effective treatments for CCA.

Although CCA is considered a malignant tumor arising from cholangiocyte transformation, recent studies utilizing genetic lineage tracing have revealed that CCA appears to originate from three types of cells: bile duct epithelial cells, hepatocytes, and hepatic stem cells [8,9,10]. To uncover the mechanisms of CCA development and its origin, various models, including carcinogen-induced and genetically modified rodent models and xenograft tumors, have been established [11,12]. These models have revealed the pathogenesis of CCA, although each model has distinct advantages and disadvantages. 

In cancer studies, tumor graft models are commonly utilized. These are useful for investigating the efficacy of novel therapeutic agents with regard to disease progression, in terms of feasibility, reproducibility, and cost and time effectiveness. While researching CCA, various xenograft and allograft models have been reported, including xenografts using CCA cells, patient-derived xenografts, and xenografts using CCA organoids [11]. The disadvantages of tumor graft models include using an immune-compromised host and a species mismatch between the host and the tumor. The genetically engineered models of CCA have been mainly reported in transgenic mice. When we are investigating a disease, including cancer, animals with genetic modifications play important roles in elucidating the complicated pathology, molecular mechanisms, and specific genes involved in disease onset and suppression. This is also true for CCA and a number of genetically engineered CCA models have been generated to try to recapitulate the specific gene mutations observed in human CCA [11]. Genetically engineered mouse CCA models have been reported to show a relatively shorter tumor development time and to recapitulate human CCA with alterations to the cellular proliferation signaling pathways, such as PI3K/AKT. These models, however, were known to frequently coexist in the development of HCC [12]. Chemically induced rodent CCA models have also been reported. Although they have been used for a long time, several of them require considerable technical skills and show a longer duration for tumor development, a relatively lower incidence, and a coincidence with HCC as well [11].

We have previously investigated a mouse colorectal tumorigenesis model and reported the use of several chemopreventive agents for colorectal cancer [13,14]. In this model, which employed C57BLKS/J-db/db (db/db) mice displaying obesity and steatohepatitis and used a colonic carcinogen azoxymethane (AOM), colorectal tumors and cancerous lesions of bile ducts in the liver were observed coincidentally. Obesity and a fatty liver have been reported to increase the risk of developing CCA [15]; therefore, we hypothesized that AOM could induce tumorigenesis of intrahepatic CCA based on the background of steatohepatitis in db/db mice.

The present study was conducted to confirm the reproducibility of the AOM-induced CCA in db/db mice and compare tumorigenesis between db/db and wild-type mice. Additionally, we analyzed the model characteristics and differences from previously reported rodent models. This novel mouse model may provide insights into the pathogenesis of intrahepatic CCA and contribute to the establishment of new therapies for this disease.

## 2. Results

### 2.1. General Observations

**Figure 1:**
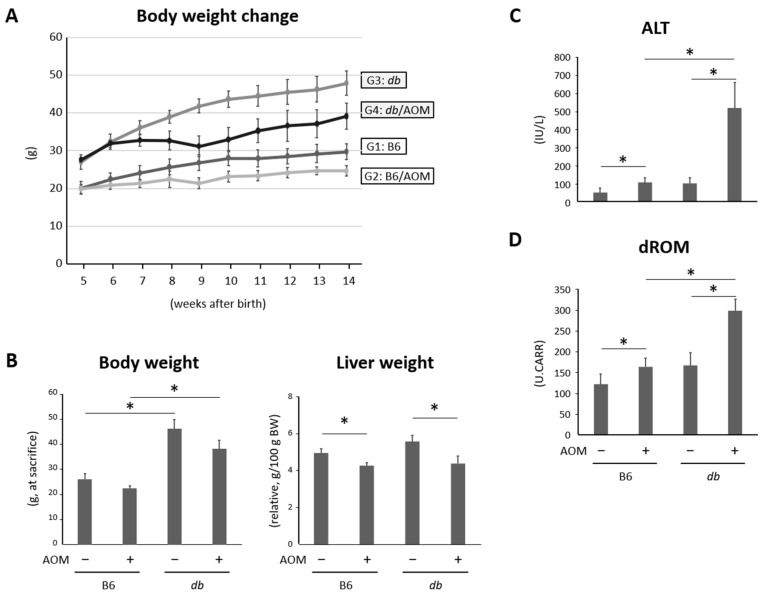
Body weight change, relative weights of liver, and serum levels of ALT and dROM. (**A**) Body weight change of mice during the experiment. (**B**) Body weights and relative weights of livers of the experimental mice at the end of study. Serum levels of (**C**) ALT and (**D**) oxidative stress marker dROM (*n* = 6). Data are the means and standard deviations. * *p* < 0.05. ALT—alanine aminotransferase; AOM—azoxymethane; B6—C57BL6/J mice; db/db—C57BLKS/J-db/db mice; dROM—derivatives of reactive oxygen metabolites.

The body weight changes and relative liver weights of the mice in all groups at the end of the study are shown in Figure 1A,B. As expected, the final body weight of the db/db mice was higher than that of the control mice. The number of mice treated with the AOM tended to be lower than that of the control group, but the difference was not significant. The relative liver weights of the mice treated with AOM were lower than those of the mice without the AOM treatment. 

### 2.2. Liver Damage and Oxidative Stress Were Observed in Mice Given Carcinogens and Were Exacerbated in Obese Mice

The serum alanine aminotransferase (ALT) levels of mice treated with carcinogens were markedly higher than those of the control mice. Comparing lean mice and obese mice with AOM, we found that the ALT levels were significantly elevated in the latter group (Figure 1C). In addition, the serum levels of derivatives of reactive oxygen metabolites (dROMs), a marker of systemic oxidative stress, were markedly higher in the AOM-administered mice than those without AOM. According to the comparison of dROM levels between the AOM-administered lean and obese mice, the oxidative stress induced by the AOM was significantly enhanced in the obese mice (Figure 1D).

### 2.3. Obese Mice Given Carcinogens Showed Extended Bile Canalicular Epithelial Hyperplasia with Atypia and Cancerous Lesions of the Bile Duct in the Liver

**Figure 2:**
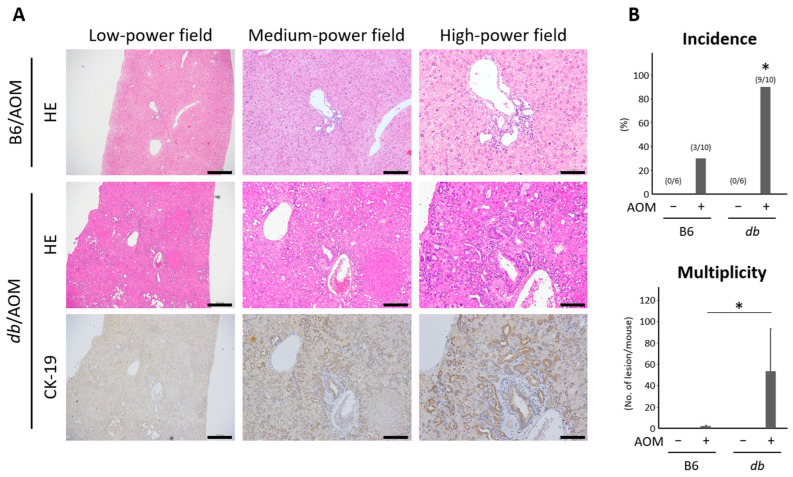
Development of hyperplasia with atypia and cancerous lesions of bile duct in the livers of mice administered with AOM. (**A**) Histopathological findings in the livers of mice, C57BL/6 and db/db, treated with AOM. HE-stained sections and immunohistochemistry for CK-19. Bars, left panels—500 μm; center panels—200 μm; right panels—100 μm. (**B**) Incidence and multiplicity of tumors. Data are the means and standard deviations. * *p* < 0.05. AOM—azoxymethane; B6—C57BL6/J mice; db—C57BLKS/J-db/db mice; HE—hematoxylin and eosin.

To investigate the reasons for liver damage in mice and to confirm the histological findings are consistent with our previously obtained experimental results, histopathological analyses for the liver of the experimental mice were performed. As expected, the obese mice showed marked steatosis in the liver in comparison with the control (Figure S1A). Intriguingly, bile canalicular epithelial hyperplasia and atypical biliary duct epithelium in the liver were observed in both the AOM-administered lean and obese mice (Figure 2A). Those hepatic lesions were stained with CK-19 (Figure 2B), and a part of the atypical biliary ducts showed a high nuclear/cytoplasm ratio and finely granular chromatin in the AOM-treated obese mice (Figure 2A and Figure S1B); this indicated that the hepatic lesions were cancerous and that they could be considered to be intrahepatic cholangiocarcinoma. The incidence, multiplicity, and area of the hepatic lesion were significantly higher in the AOM-administered obese mice compared to the control (Figure 2B). 

### 2.4. Hepatic Expression Levels of Genes as Markers to Investigate the Type of Cancerous Lesions Were Analyzed by Microarrays and a qRT-PCR

**Figure 3:**
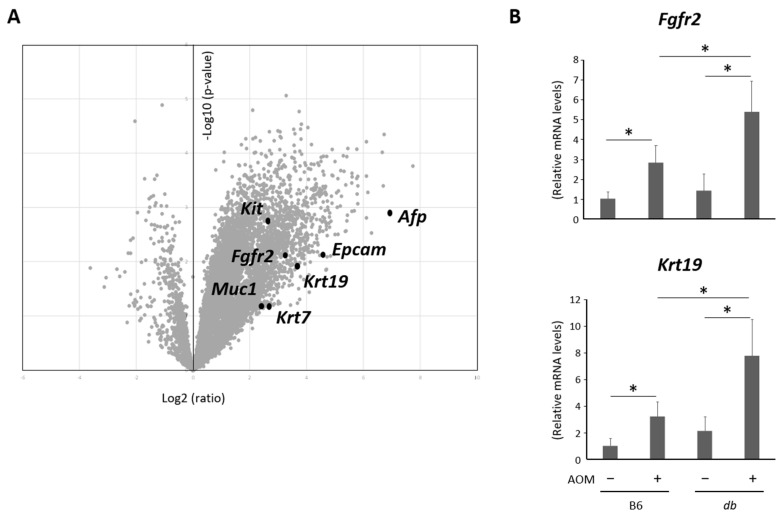
Analyses of microarray and gene expressions in the liver of the experimental mice. (**A**) Volcano plot of microarray data showing differentially expressed genes between obese mice with and without AOM administration. The plot displays the ratio (*x*-axis) and the significance (*y*-axis) of the identified genes. The ratio and the significance are converted to Log2(ratio) and −Log10(*p*-value), respectively. Black dots indicate several genes of interest (not all). (**B**) The expression of several of these genes was validated using qRT-PCR (*n* = 6). * *p* < 0.05. AOM, azoxymethane; B6, C57BL6/J mice; db/db, C57BLKS/J-db/db mice.

To further analyze the bile duct cancerous lesions, microarray analyses were performed by comparing liver samples from the untreated and the AOM-administered obese mice. Since volcano plots revealed that a number of genes were up- or down-regulated in the AOM-treated group compared to the control, we focused on several altered genes related to the type of cancerous lesions (Figure 3A). The findings indicated that the levels of the genes reported as phenotypic markers for cholangiocarcinoma, including *Epcam*, *Kit*, *Krt7*, *Krt19*, and *Muc1* [16,17,18,19,20], were upregulated, suggesting that the cancerous lesions in the liver appeared to be cholangiocarcinoma. In addition, there was an up-regulated gene expression of *Fgfr2*, which is reported to be correlated with FGFR2 fusion is detected in cases of CCA. [21,22]. On the other hand, *Afp*, a marker for HCC, was also exhibited elevated gene expression. This suggests that tiny cancerous and precancerous lesions originating from hepatocytes may exist, although these lesions could not be detected via a microscopic investigation. We then evaluated the expression of several genes, including *Fgfr2* and *Krt19*, using a quantitative real-time reverse transcription-PCR (qRT-PCR), revealing that these genes were markedly up-regulated in the AOM-administered mice compared to their controls. In addition, these gene expressions were significantly elevated in the AOM-treated obese mice compared to those in the AOM-treated lean mice (Figure 3B). 

### 2.5. Expression Levels of Genes Related with Inflammation Were Elevated in the Liver of Obese and AOM-Treated Mice

**Figure 4:**
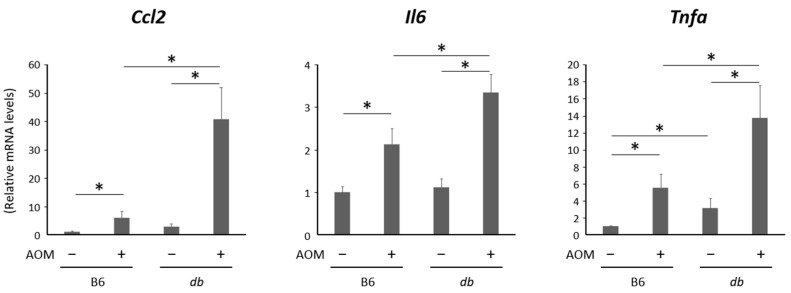
Relative expression levels of genes related to inflammation in the liver of experimental mice. mRNA expression levels in liver were evaluated among all groups using qRT-PCR (*n* = 6). Data are the means and standard deviations. * *p* < 0.05. AOM—azoxymethane; B6—C57BL6/J mice; db/db—C57BLKS/J-db/db mice.

As hepatic damage and inflammation may contribute to the development of biliary duct lesions, inflammatory markers in the liver were investigated. The expression levels of genes for the pro-inflammatory markers, *Ccl2*, *Il6*, and *Tnfa*, were analyzed applying a qRT-PCR using the liver samples, which revealed that these markers were significantly up-regulated in the AOM-administered mice compared to their controls (Figure 4). The gene expression of *Tnfa* in the obese mice without AOM was markedly increased compared to that in the lean mice. Moreover, the expressions of the above genes were significantly elevated in the AOM-treated obese mice compared to those in the AOM-treated lean mice. These results suggest that, in addition to the chronic inflammation caused by steatohepatitis, carcinogen administration causes bile duct hyperplasia, which might lead to atypical epithelial formation and carcinogenesis in the biliary ducts in the liver. The serum levels of these cytokines were expected to vary similarly to the hepatic gene expressions, but in this study the measurement was not performed because hepatic inflammation was focused, which is known as the precedent in human CCA. 

## 3. Discussion

CCA is the most common biliary tract cancer and the second most common primary cancer in the liver after HCC [1]. While CCA is relatively rare, this malignancy is considered aggressive, with a poor prognosis [3,4]. Recently, several molecular targeted drugs have been developed for CCA [5]; however, there is still a need to establish effective treatments for this disease. Previous reports have described various animal models of CCA, including carcinogen-induced and genetically engineered rodent models and xenograft tumors [11,12]. 

In this study, we developed a novel mouse model of AOM-induced malignant lesions in the biliary ducts, particularly in obese db/db mice. The bile duct lesions were histopathologically considered cancerous, and an analysis of molecular markers indicated that the lesions presumably appeared to be malignant in the biliary tracts of the liver; however, metastases or invasions were unconfirmed. These findings indicate that this is a novel intrahepatic CCA mouse model, and this report is the first to demonstrate the use of AOM for the development of CCA in mice. Using another type of animal, methylazoxymethyl acetate, a derivative of AOM, was also reported to induce cholangiocarcinoma in hamsters [23]. Recently, selective FGFR inhibitors have been approved as molecular targeted drugs that can be used for the treatment of FGFR2 fusion- or rearrangement-positive CCA [24]. FGFR2 fusion was detected at a significantly higher rate than in HCC, and FGFR2 fusion-positive CCA exhibited increased expression of *Fgfr2* mRNA [21,22]. In the present study, a histopathological analysis and the upregulated expression levels of biliary markers and *FgfR2* led to a diagnosis of CCA.

Several rodent CCA models have been reported and are considered to have both strengths and weaknesses. Genetically engineered mouse models of CCA, such as *Alb-Cre:Kras^G12D^:p53^−/−^* and *Alb-Cre:Kras^G12D^:Pten^−/−^*, reportedly exhibit a relatively shorter tumor development duration and recapitulate human CCA with PI3K/AKT pathway alterations; however, these models frequently coexist in the development of HCC [25,26]. Other reported genetically engineered mouse CCA models included mice with constitutive expressions of the human epidermal growth factor receptor 2 (ERBB2), mice with hepatocyte-specific disruption of tumor suppressors SMAD4 and PTEN, carbon tetrachloride-administered p53 knockout mice, and mice with hepatocyte-specific constitutive Notch1 expression [27,28,29,30]. Recently, hepatocyte-specific TNF receptor-related factor 3- and PTEN-deficient mice have also been reported [31].

Rodent models of carcinogen-induced CCAs have also been studied. Dimethylnitrosamine (DMN)- or diethylnitrosamine (DEN)-based CCA models have been used for a long time and have been reported to show a relatively lower tumor incidence and coincidence with HCC [32,33]. Indeed, we have previously reported that DEN administration led to the development of HCC in C57BL/6 mice, and the incidence of HCC was above 80% [34]. In combination with DMN or DEN and bile duct ligation (BDL), there is a relatively higher incidence of CCA and a shorter duration for tumor development, but the models require significant technical skills for BDL [35]. Thioacetamide (TAA) is a chemical compound frequently used to induce CCA in rats; however, the detailed mechanism of carcinogenesis remains unknown. This model exhibits high reproducibility for the development of CCA or premalignant dysplastic lesions of the bile duct without complicated genetic engineering or considerable technical skills; however, tumor development requires a longer duration (22–24 weeks) [36].

Similar to previously reported rodent models for CCA, our mouse model also has advantages and disadvantages. The duration to tumor development in our model is 12 weeks, which is relatively short. The abovementioned DMN or DEN plus BDL and TAA models required more than 22 weeks of treatment [35,36]. While the genetically modified mouse models showed a shorter duration of tumor development than our model, these mice exhibited growth disorders and short lives [26]. The model in this study required only four intraperitoneal injections of the AOM with 90% tumor incidence. In addition, this model utilizes only commercially available mice that have not been genetically engineered and does not require advanced technical skills. Moreover, cancerous lesions in this model developed in the background of a liver injury and inflammation, which are frequent precedents in human CCA. Table S1 provides a summary of previous reports and the present study on animal CCA models.

There are disadvantages to the proposed CCA model. First, we observed no obvious metastases or invasions in the mice during the experimental period; therefore, it was difficult to determine whether the lesion was malignant. Second, the model developed CCA and might have been accompanied by HCC according to the results of the microarray analyses, although HCC was not clearly detected using microscopy. Among the previously reported CCA models [11], only a few showed metastases and invasion, and many of them showed concurrent HCC development; therefore, these disadvantages may not mean that our mouse model is inferior to others. Regarding the concurrence of other cancers, the mouse model originally exhibited premalignant lesions in the colorectum. These colorectal lesions are thought to be precursors and might have little considerable effect on the whole body status as well as the disease state of CCA in the liver [13,14]. Considering the limitations of this study, whole liver samples were used instead of cancerous lesions of the biliary duct to investigate phenotypic markers for CCA using microarray and qPCR analyses. Cell-specific analysis via microdissection was not performed in this study, which may have led to precise or different findings. In addition, it is also suggested that bile duct lesion-specific analyses provide novel findings of effective biomarkers for CCA. Further investigations, including the above-mentioned ones, need to be carried out to reveal the characteristics of the mouse CCA model.

When providing animal models, their usefulness for the evaluation of therapeutic agents needs to be considered. The advantages of our model for therapeutic approaches are thought to include using a commercially available mouse, a shorter experimental duration for tumor development, higher tumor incidence, and a longer expected life span. In terms of life span, genetically engineered models were reported to exhibit growth disorders and shortened (by a few months) lifetimes (Table S1). Although we did not check the natural lifetime of our mouse CCA model, neither growth disorder nor severe weight reduction were observed, at least during the experiment; therefore, a longer life span was expected compared to the above-mentioned models. This point must be of importance for evaluating potential therapies. If rodents die in a short time due to the disease, it is impossible to assess disease activity or tumor shrinkage. It would be difficult to evaluate the efficacy of potential therapies based on only days of survival in experimental rodents. The disadvantages of our present model are considered to include the absence of metastases and an inability to see the mass of CCA macroscopically. The cancerous lesions of our CCA model were observed microscopically, and this could make evaluation of drug efficacy difficult. Another disadvantage of our model is thought to be that it employs obese mice. The mice used in our model show metabolic disorders, such as diabetes, dyslipidemia, and fatty livers. These factors may affect the efficacy of potential therapies because of differences in fluid composition and metabolic enzymes; therefore, the pharmacodynamics, efficacy, and toxicity of potential medications may not be accurately evaluated.

The possible mechanisms underlying CCA development in this mouse model can be explained. The carcinogen AOM used in the present study induces DNA damage, leading to KRAS mutations and subsequent tumor development [37]. Although AOM is known as a rodent colon-specific carcinogen, KRAS mutations have been detected in the neoplasia of the biliary epithelium and intrahepatic CCA [38]. Therefore, AOM might cause DNA damage in the biliary ducts as well, leading to CCA development. As CCA has been reported to develop in the context of hepatic damage and chronic inflammation, steatohepatitis in the present mouse model may have contributed to tumor promotion. In addition, oxidative stress induced by reactive oxygen species (ROS) is known to induce DNA damage and hepatotoxicity [39,40], which are enhanced in obese mice administered AOM, and might also promote tumor development. Interestingly, the caloric homeostasis-regulating hormone leptin has been reported to significantly affect the development of CCA [41]. In the present study, tumor formation was significantly promoted in the db/db mice, which showed leptin receptor dysfunction and elevated serum leptin levels [42], suggesting that using db/db mice is the most important point in our model. Further studies should be performed to investigate whether leptin is a key factor in this CCA model by blocking leptin signaling in obese mice and conducting leptin administration in lean mice.

## 4. Materials and Methods

### 4.1. Animals, Chemicals, and Diets

Male db/db and control C57BL6/J mice were purchased from Japan SLC, Inc. (Shizuoka, Japan). They were kept at the Gifu University Animal Facility under controlled conditions of humidity (50 ± 10%), light (12/12 h light/dark cycle), and temperature (23 ± 2 °C), according to the institutional animal care guidelines. All mice were housed in plastic cages with free access to drinking water and a basal diet of CRF-1 (Oriental Yeast Co., Ltd., Tokyo, Japan). The AOM was purchased from Wako Pure Chemical Co. (Osaka, Japan). All methods were reported in accordance with the ARRIVE guidelines (https://arriveguidelines.org, accessed on 1 May 2022) and performed in accordance with the relevant guidelines and regulations.

### 4.2. Experimental Procedure

At five weeks of age, the 16 db/db and the 16 control mice were quarantined for the first seven days and then separated into four groups. Six and 10 control mice were assigned to groups 1 and 2, respectively. Six and 10 db/db mice were assigned to groups 3 and 4, respectively. From six weeks of age, mice in groups 2 and 4 received a subcutaneous injection of AOM (15 mg/kg body weight) once a week for 4 weeks. This regimen of AOM injection was referred to in previous reports [13,14], in which the AOM was originally used as a colonic carcinogen. The mice in groups 1 and 3 received a subcutaneous injection of saline as a control once a week for 4 weeks. At the end of the study (18 weeks of age), all mice were sacrificed by CO_2_ asphyxiation in accordance with a previous report [43]. All experimental procedures were approved by the Committee of Institutional Animal Experiments at Gifu University (authorization code 2022-081 on 23 May 2022).

### 4.3. Blood Biochemistry

Whole blood was collected from the inferior vena cava and centrifuged to obtain the serum for chemical analyses. Serum ALT levels were determined in a commercial laboratory (SRL Inc., Tokyo, Japan). The dROM was determined as a marker of oxidative stress and measured using the FREE Carpe Diet (Diacron International s.r.l., Grosseto, Italy).

### 4.4. Histological Analysis and Immunohistochemistry

For histological evaluation, the livers were fixed in 10% buffered formalin, embedded in paraffin, and stained with hematoxylin and eosin (HE). Hepatic lesions were diagnosed by two pathologists evaluating nuclear/cytoplasm ratio and granular chromatin of the bile duct from HE-stained tissue with reference to the previous reports [25,26]. The incidence and multiplicity were evaluated based on the presence of hepatic biliary lesions including cancerous and atypical bile ducts, because biliary lesions in the liver were not clearly distinguished into cancer or severe atypia. Immunohistochemical staining was performed for CK-19 (ab15463; Abcam, Cambridge, UK).

### 4.5. RNA Extraction and Quantitative Real-Time Reverse Transcription-PCR Analysis

Total RNA was isolated from the whole liver samples of the experimental mice using an RNeasy Mini Kit (QIAGEN, Venlo, The Netherlands). cDNA was synthesized from the total RNA using a high-capacity cDNA Reverse Transcription Kit (Applied Biosystems, Foster City, CA, USA). A qRT-PCR analysis was performed using the LightCycler 96 System (Roche Diagnostics, Indianapolis, IN, USA) with LightCycler 480 SYBR Green I Master Mix (Roche Diagnostics). The specific primers used to amplify *Ccl2*, *Fgfr2*, *Il6*, *Krt19*, *Tnfa*, and *18s* genes were obtained from previous reports [14,44,45] or designed using Primer-BLAST (https://www.ncbi.nlm.nih.gov/tools/primer-blast/, accessed on 1 March 2023) as follows: *Ccl2*, forward 5′-CCA TCA GTC CTC AGG TAT TGG-3′, reverse 5′-CTT CCG GAC GTG AAT CTT CT-3′; *Fgfr2*, forward 5′-CAA ACA CTC GTC CCC TGT CT-3′, reverse 5′-GGA TGT TCG TGG GAG ATG TT-3′; *Il6*, forward 5′-CCG GAG AGG AGA CTT CAC AGA G-3′, reverse 5′-CTG CAA GTG CAT CAT CGT TGT T-3′; *Krt19* forward 5′-CCA TCT GAG CTA CCA GCG AG-3′, reverse 5′-GTC GAG GGA GGG GTT AGA GT-3′; *Tnfa*, forward 5′-TGG CCC AGA CCC TCA CAC TCA G-3′, reverse 5′-ACC CAT CGG CTG GCA CCA CT-3′; and *18s*, forward 5′-CCA TCC AAT CGG TAG TAG CG-3′, reverse 5′-GTA ACC CGT TGA ACC CCA TT-3′. The expression levels of these genes were normalized to those of *18s*. The expression levels of the mRNA were measured in triplicate.

### 4.6. Microarray Analysis

As described above, total RNA was extracted from the whole liver samples, and RNA quality was checked (concentration > 0.5 µg/µL and OD260/280, 1.8–2.0). A cDNA microarray analysis was conducted using a 3D-GeneTM Mouse Oligo Chip 24k (Toray Industries, Tokyo, Japan). The microarray slides were scanned using a 3D-Gene Scanner (Toray Industries) and processed using the 3D-Gene Extraction software version 2.0.0.6 (Toray Industries). The results of microarray analysis were represented using a volcano plot [46], where the horizontal axis was the expression ratio (Log2) and the vertical axis was the *p*-value (−Log10), comparing liver samples from the untreated and AOM-administered obese mice.

### 4.7. Statistical Analyses

One-way or two-way analyses of variance (ANOVA) were used to compare specific groups after the Shapiro–Wilk normality test, which was followed by the Tukey–Kramer multiple comparison test to confirm statistical significance. For non-parametric statistical analysis, a Kruskal–Wallis test and Steel–Dwass tests were performed. Fisher’s exact test was used to compare the incidence of biliary duct lesions. Data are presented as the mean ± standard deviation, and the statistical significance was set at *p* < 0.05.

## 5. Conclusions

In conclusion, we established a novel mouse model of intrahepatic CCA in obese db/db mice administered with AOM. While the model did not require complicated genetic modifications or technical skills, it showed a markedly high tumor incidence with a short experimental duration in a background of steatohepatitis. These findings and further intensive investigations of this model will help elucidate the pathogenic mechanism of CCA and establish novel preventive and therapeutic strategies for this disease.

## Data Availability

The data presented in this study are available on request from the corresponding author.

## Supplementary Materials

The following supporting information can be downloaded at: https://www.mdpi.com/article/10.3390/ijms241914581/s1.
