# Supplementary material for: A Novel Mouse Model of Intrahepatic Cholangiocarcinoma Induced by Azoxymethane
Source: Int J Mol Sci. 2023 Sep 26;24(19):14581. doi: 10.3390/ijms241914581 (PMC10572168; doi:10.3390/ijms241914581)
Supplement: Supplementary file 1 [file ijms-24-14581-s001.zip › ijms-2556416-supplementary.pdf]

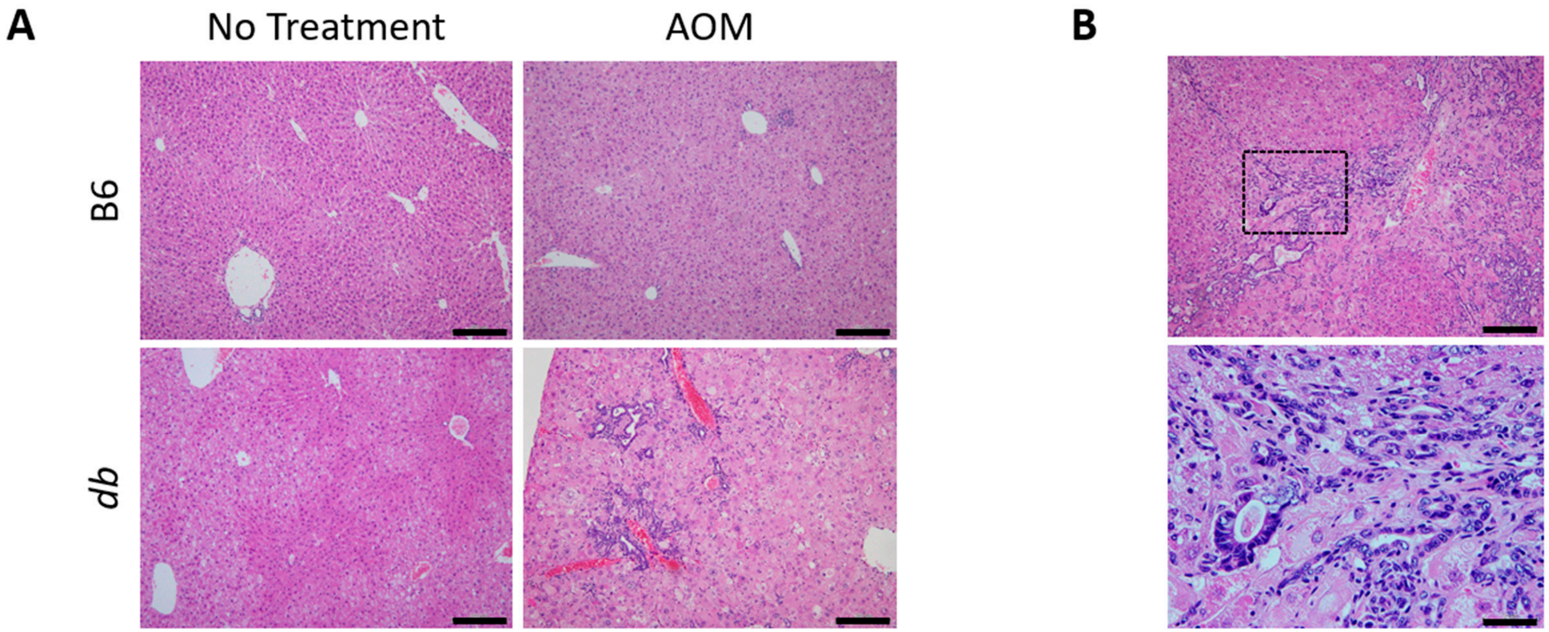

Figure S1. (**A**,**B**) Histopathological analyses for the liver of the experimental mice.

Table S1. Summary for previous reports and present study of rodent CCA models.

Model (chemical or genetic modification)	Animal	Type of lesion	Time for tumor development	Yield	Characteristics	Reference
DEN and BDL	Mouse	CCA	28 weeks	50%	- Need technical skills for BDL<br>- Need longer time	[35]
Alb-Cre:Kras^{G12D}*:p53*$^{-/-}$	Mouse	CCA	9 weeks	83%	- Concurrence of HCC<br>- Mean life span was 19 weeks<br>- Metastatic tumor	[25]
Alb-Cre:Kras^{G12D}*:Pten*$^{-/-}$	Mouse	CCA	8 weeks	Not indicated	- Growth disorder<br>- Median survival was 46 days	[26]
TAA	Rat	Cholangiocyte dysplasia - CCA	24 weeks	100%	- Using rats<br>- Need longer time	[36]
AOM (present study)	Mouse	Atypical bile duct - CCA	12 weeks	90%	- Using db/db mice<br>- No genetic modification	N/A

AOM, azoxymethane; BDL, bile duct ligation; CCA, cholangiocarcinoma; DEN, diethylnitrosamine; HCC, hepatocellular carcinoma; TAA, thioacetamide.
